# Seroprevalence of anti-SARS-CoV-2 antibodies and factors associated with infection among adolescent men who have sex with men and transgender women in Salvador, Brazil

**DOI:** 10.1186/s12889-022-14969-x

**Published:** 2023-01-09

**Authors:** Carina C. Santos, Fernanda W. de M. Lima, Laio Magno, Fabiane Soares, Dulce Ferraz, Alexandre Grangeiro, Eliana Miura Zucchi, Marie Préau, Xavier Mabire, Helen R. S. S. Matos, Inês Dourado, the COBra Study Group

**Affiliations:** 1 Departamento de Análises Clínicas e Toxicológicas, Faculdade de Farmácia, Universidade Federal da Bahia, Salvador, Brazil; 2 Departamento de Ciências da Vida, Universidade do Estado da Bahia, Salvador, Brazil; 3 Instituto de Saúde Coletiva, Universidade Federal da Bahia, Salvador, Brazil; 4 Escola FIOCRUZ de Governo, Fundação Oswaldo Cruz, Brasília, Brazil; 5 Pole of Social Psychology, UMR 1296 Radiations: Défense, Santé, Environnement, Université Lyon 2, Lyon, France; 6 PHASE (Psychology of Health, Aging and Sport Examination), Faculty of Social and Political Science, University of Lausanne, Lausanne, Switzerland; 7 Faculdade de Medicina, Universidade de São Paulo, São Paulo, Brazil; 8 Programa de Pós-Graduação em Saúde Coletiva, Universidade Católica de Santos, Santos, Brazil; 9 Institut de Psychologie, Université de Lausanne, Lausanne, Switzerland

**Keywords:** COVID-19, Antibodies, Prevalence, Men who have sex with men, Transgender women, Adolescent, Brazil

## Abstract

**Background:**

Brazil was strongly affected by the COVID-19 pandemic and the impact of the pandemic on sexual and gender minorities’ youth remains unknown. This study aimed to estimate the seroprevalence of SARS-CoV-2 antibodies and associated factors among adolescent men who have sex with men (AMSM) and transgender women (ATGW) participants of a human immunodeficiency virus (HIV) pre-exposure prophylaxis cohort study (PrEP1519).

**Methods:**

This is a cross-sectional design conducted between June and October 2020 in Salvador, Brazil. Serum samples were collected from AMSM and ATGW aged 16-21 years between June-October 2020. IgG and IgM anti-SARS-CoV-2 were detected by chemiluminescence immunoassay, and data were collected through a socio-behavioral questionnaire.

**Results:**

Among the 137 participants, the seroprevalence of anti-SARS-CoV-2 IgG and IgM was 20.4%; 16.8% of the participants were positive for IgG, and 11.7% for IgM. In the multivariable analysis, the seroprevalence was two times higher among those who never wore masks (OR= 2.22; 95% CI: 1.08-4.57) and among those who believed that they could be easily cured of the disease (OR= 2.05; 95% CI: 1.05-4.01).

**Conclusions:**

The high seroprevalence of SARS-CoV-2 antibodies among gender and sexual minority youth seems to be informed by behaviors and attitudes that contrast with public health measures and the potential severity of the disease when vaccination was still not available.

## Background

Since the first case of coronavirus disease 2019 (COVID-19) identified in Brazil, on February 26, 2020, more than 30 million cases and 670,000 deaths have been reported [1]. Brazil is among the five countries with the highest number of deaths and cases in the world [2] despite having one of the lowest proportions of COVID-19 diagnostic tests [3], suggesting a high underreporting rate.

Notwithstanding the improvement in the epidemiological situation with the vaccine rollout in Brazil and the world, the global state of emergency caused by the severe acute respiratory syndrome coronavirus 2 (SARS-CoV-2) remains [4]. The dynamics of the pandemic has varied around the world with populations facing different “waves” [5]. More than 500 million cases have been confirmed, including more than 6 million deaths worldwide [6].

Vulnerable population groups have been more severely impacted by the pandemic [7, 8]. For example, men who have sex with men (MSM) and transgender women (TGW) are sexual or gender minority groups and historically marginalized whose lifestyle and social conditions may be associated with an increased risk of SARS-CoV-2 infection [9–11]. Studies have indicated that adult MSM in Brazil, during the first year of the pandemic (2020), had difficulty complying with social isolation measures and engaged in sexual encounters with multiple partners [12–14]. Among adolescent MSM and TGW, it has been shown that the pandemic disrupted important dimensions of their life conditions and affected sexual and preventive behaviors [15]. Another study conducted with TGW in India, highlighted the lack of adequate knowledge, perceptions of low risk and non-adherence to COVID-19 prevention measures, and fear of declaring disease-related symptoms [9].

To date, few studies have analyzed the impact of the SARS-CoV-2 pandemic on these specific populations and more specifically among adolescents. Accordingly, this study aimed to estimate the seroprevalence of specific antibodies against SARS-CoV-2 and its associated factors among adolescent MSM (AMSM) and TGW (ATGW) participants of an HIV pre-exposure prophylaxis demonstration study (PrEP1519).

## Methods

### Study design and population

This is a cross-sectional design conducted before COVID-19 vaccination was available, and used data from the ANRS-COBra Study, a mixed-method study [16] nested in PrEP1519, an ongoing demonstration study of the effectiveness of PrEP conducted in three Brazilian cities – Salvador, Belo Horizonte, and São Paulo – from 2019 to 2021. Inclusion criteria in the cohort were AMSM and ATGW aged 15–19 years at enrollment, who had at least one sexual intercourse with another cisgender man or transgender woman and reported spending most of their time in the cities where the study takes place (i.e., living, studying, working, or residing in one of the study sites). After the eligibility criteria check and provision of information on the proposed steps for the study, those who agreed to participate provided written informed consent or assent. This study (PrEP1519) was approved by the Research Ethics Review Committee (ERC) of the World Health Organization (Protocol ID: Fiotec-PrEP Adolescent study), the Federal University of Bahia (# 3,224,384), and of the University of São Paulo (USP) (Protocol ID: #3,082,360). During the COVID-19 pandemic, we submitted a COVID-19 testing amendment to the USP ERC and it was approved (Protocol ID: #4,229,488).

Participants were offered PrEP and other combination prevention methods and a range of sexual health care. Follow-up procedures include quarterly visits to the healthcare facilities for medical consultations, counseling, and HIV and other sexually transmitted infection (STI) testing [17]. During the COVID-19-related quarantine period, several of these consultations and procedures were done through telehealth, using videoconference, texting, and mailing PrEP pills and HIV self-tests [18].

### Data collection and detection of anti-SARS-CoV-2 antibodies

To estimate the SARS-COV-2 prevalence and associated factors to the infection, data was collected from a sample of 137 participants of the PrEP1519-Salvador site aged 16-21 years (as the cohort ages).

A socio-behavioral questionnaire with questions about gender identity, access to health services, sexual practices, drug and alcohol use, and history of violence was applied at the PrEP1519 cohort baseline and quarterly visits. Furthermore, between June and October 2020 participants were also invited to answer socio-behavioral with questions concerning their experiences during the pandemic. The questionnaire could be accessed through a link sent via an instant messaging application and included questions on sociodemographics, behaviors during the pandemic, knowledge about SARS-CoV-2 transmission and prevention measures, and perceptions of the pandemic (COBra questionnaire). For quality control, one of the field researchers double-checked all the participants’ answers.

In the follow-up visits of the PrEP1519 participants, blood samples were collected specifically for anti-SARS-CoV-2 IgM and IgG antibodies detection from June to October 2020. Whole blood was collected from the cubital vein by a trained phlebotomist into a serum separator clot activator tube (BD Vacutainer® SST ™ - Yellow Top). After the collection, the samples were centrifuged at 3000 rpm for 10 minutes, at room temperature, and the serum was separated and stored at -20 ° C (freezer) until the testing procedures. Anti-SARS-CoV-2 IgM and IgG antibodies were detected by chemiluminescence using the SARS-CoV-2 IgG and SARS-CoV-2 IgM kits (Abbott Diagnostics), according to the manufacturer’s instructions. The results were assessed on an ARCHITECT i1000SR immunoassay analyzer (Abbott Diagnostics).

### Study variables

The outcome variable was the anti-SARS-CoV-2 IgG or IgM seropositivity. Other study variables were organized into six groups: i. sociodemographic: age (16-18; 19-21 years), population group (AMSM; ATGW); ii. household characteristics: number of people in the household (≤ 3 people; > 3 people), living alone (no; yes), living with mother (no; yes), living with siblings (no; yes), number of rooms at home (≤ 3 rooms; > 3 rooms); iii. access to healthcare services (exclusively through the Brazilian National Health System; in Portuguese *Sistema Único de Saúde* – SUS); exclusively through a private health insurance plan; direct payment for consultations; and mixed – i.e., all of the above); iv. adherence to SARS-CoV2 prevention measures: quarantine (no; yes), wearing masks in public places (no; yes), frequent hand washing (no; yes); v. perceptions about COVID-19: SARS-CoV-2 infection risk perception (low; high), perception of severity (low; moderate and very severe), believing that COVID-19 can be easily cured (no; yes), self-reported infection with SARS-CoV-2 (not known; yes, but without confirmatory tests; yes, confirmed by a test of clinical diagnosis); vi. behaviors during quarantine: alcohol use (no; yes, and with increased frequency; yes, and with the same frequency) and sexual partner during the pandemic (no; yes, with a steady partner; yes, with casual partners; yes, with steady and casual partners).

### Data analysis

A descriptive analysis of the study population characteristics was carried out. Bivariate analysis to estimate the seroprevalence of anti-SARS-CoV-2 IgG or IgM antibody by study variables was conducted using the Chi-squared test or Fisher’s exact test. A logistic regression model was performed to estimate adjusted odds ratios for the multivariable analysis. Posteriorly, based on this model, adjusted prevalence ratios (aPR) and respective 95% confidence interval (95%CI) of the association between study variables and SARS-CoV-2 infection were estimated using marginal standardization. The delta method is applied to obtain standard errors [19, 20]. The variables with *p*-value ≤ 0.20 in the bivariate analysis were selected to start modeling and only those with *p*-value < 0.05 remained in the final model, using a backwards procedure. Hosmer-Lemeshow test was used to assess the fit of the final model [21]. The analysis was conducted using the software STATA version 17.0 (StataCorp, 2015).

## Results

In terms of sociodemographic characteristics, most study participants were 19 to 21 years old (66.4%), AMSM (81.7%), lived with their mother (64.2%), and in homes with more than three rooms (73.7%). A minority lived alone (2.7%) or with siblings (38.8%), and half of the participants lived with more than 3 people in the same household (50.4%). Most participants reported using SUS healthcare services exclusively (74.0%).

Although most (86.9%) reported adhering to quarantine measures, 19.1% reported never wearing masks in public places, and half reported not washing their hands frequently (48.8%). Regarding the perceptions of the COVID-19 pandemic, most participants reported a perception of a high risk of infection with SARS-CoV-2 (70.1%) and COVID-19 severity (55.7%) and did not believe that they could be easily cured of the disease (76.1%).

Regarding behavioral aspects during quarantine, one-fifth of the participants reported increasing the frequency of alcohol consumption (25.4%) and most reported having had sex with casual and/or steady partners (67.2%) (Table 1).

**Table 1:** Sociodemographic, behavioral characteristics and perceptions about the COVID-19 pandemic among AMSM and ATGW in the PrEP1519 cohort, Salvador, Brazil, 2020.

**Variables**	**n**	**%**
**Sociodemographic**		
**Age**		
16-18 years	46	33.6
19-21 years	91	66.4
**Population group**		
ATGW	24	18.3
AMSM	107	81.7
**Household characteristics**		
**Number of household members**		
≤ 3 people	68	49.6
> 3 people	69	50.4
**Living alone**		
No	131	97.8
Yes	3	2.2
**Living with the mother**		
No	48	35.8
Yes	86	64.2
**Living with siblings**		
No	82	61.2
Yes	52	38.8
**Number of rooms at home**		
>3 rooms	101	73.7
≤ 3 rooms	36	26.3
**Access to healthcare services**		
Exclusively SUS	97	74.0
Exclusively private health plan	9	6.9
Direct payment for consultations	1	0.8
Mixed (all of the above)	24	18.3
**Adherence to SARS-CoV-2 prevention measures**		
**Quarantine**		
No	18	13.1
Yes	119	86.9
**Using mask in public places**		
Always	106	80.9
Never	25	19.1
**Frequent hand washing**		
No	67	51.2
Yes	64	48.8
**Perceptions about COVID-19**		
**SARS-CoV-2 infection risk perception**		
Low	41	29.9
High	96	70.1
**Perception of severity**		
Low	7	5.4
Moderate	51	38.9
High	73	55.7
**Believing that COVID-19 can be easily cured**		
No	102	76.1
Yes	32	23.9
**Self-reported infection with SARS-CoV-2**		
Not known	114	87.0
Yes, but without confirmatory tests	10	7.6
Yes, confirmed by a test of clinical diagnosis	7	5.4
**Behaviors during quarantine**		
**Alcohol use**		
No	60	46.1
Yes, and with increased frequency	33	25.4
Yes, with the same frequency	37	28.5
**Sexual partner during the pandemic**		
No	43	32.8
Yes, with a steady partner	45	34.4
Yes, with casual partners	25	19.1
Yes, with both	18	13.7

The global seroprevalence of SARS-CoV-2 was 20.4% (95% CI: 14.4-28.0), and 16.8% (95% CI: 11.3-24.0) for IgG and 11.7% (95% CI: 7.2-18.2) for IgM antibodies, respectively (Table 2).

**Table 2:** Seroprevalence of SARS-CoV-2 infection among AMSM and ATGW in the PrEP1519 cohort, Salvador, Brazil, 2020.

**Variable**	**n**	**%**	**95% CI**
**SARS-CoV-2 infection (IgG+ / IgM+)**			
Positive	28	20.4	14.4-28.0
Negative	109	79.6	71.9-85.5
**IgG**			
Positive	23	16.8	11.3-24.0
Negative	114	83.2	75.9-88.6
**IgM**			
Positive	16	11.7	7.2-18.2
Negative	121	88.3	81.7-92.7

In the bivariate analysis, the SARS-CoV-2 seroprevalence was higher among adolescents who reported living with siblings (30.8%); those that never wore masks in public places (36.0%), those with a high COVID-19 risk perception (25.0%), and among those who believed that they could be easily cured of the disease (34.4%) (*p* < 0.05). In the multivariable analysis, the seroprevalence of SARS-CoV-2 was two times higher among those who never wore masks (OR= 2.22; 95% CI: 1.08-4.57) and among those who believed that they could be easily cured of the disease (OR= 2.05; 95% CI: 1.05-4.01) (Table 3).

**Table 3:** Bivariate and multivariable analysis of the association of study variables with SARS-CoV-2 infection seroprevalence among AMSM and ATGW in the PrEP1519 cohort, Salvador, Brazil, 2020.

**Variables**	**COVID-19 (IgG+/IgM+)**				
	**Bivariate analysis**			Multivariable analysis**	
**Sociodemographic**	**n/N**	**%**	***P*-value**	aPP (95% CI) ***	
**Age**			0.244		
16-18 years	12/46	26.1			
19-21 years	16/91	17.6			
**Population group**					
ATGW	5/24	20.8	0.976		
AMSM	22/107	20.6			
**Household**					
**Number of household members**			0.099		
≤ 3 people	10/68	14.7			
> 3 people	18/69	26.1			
**Living alone**			0.508*		
No	27/131	20.6			
Yes	1/3	33.3			
**Living with the mother**			0.074		
No	6/48	12.5			
Yes	22/86	25.6			
**Living with siblings**			0.025		
No	12/82	14.6			
Yes	16/52	30.8			
**Number of rooms at home**			0.863		
>3 rooms	21/101	20.8			
≤ 3 rooms	7/36	19.4			
**Access to healthcare services**			0.678*		
Exclusively SUS	19/97	19.6			
Exclusively private health plan	3/9	33.3			
Direct payment for consultations	-	-			
Mixed (all of the above)	5/24	20.8			
**Adherence to SARS-CoV-2 prevention measures**					
**Quarantine**			0.145		
No	6/18	33.3			
Yes	22/119	18.5			
**Using mask in public places**			0.034		
Always	18/106	17.0		1.00	
Never	9/25	36.0		2.22	(1.08-4.57)
**Frequent hand washing**			0.934		
No	14/67	20.9			
Yes	13/64	20.3			
**Perceptions about COVID-19**					
**SARS-CoV-2 infection risk perception**			0.063*		
Low	4/41	9.8			
High	24/96	25.0			
**Perception of severity**			0.873*		
Low	1/7	14.3			
Moderate	12/51	23.5			
High	14/73	19.2			
**Believing that they can be easily cured of the disease**			0.032		
No	17/102	16.7		1.00	
Yes	11/32	34.4		2.05	(1.05-4.01)
**Behavioral**					
**Alcohol use during quarantine**			0.478		
No	10/60	16.7			
Yes, and with increased frequency	9/33	27.3			
Yes, albeit with the same frequency	8/37	21.6			
**Sexual partner during the pandemic**			0.064*		
No	9/43	20.9			
Yes, with a steady partner	7/45	15.6			
Yes, with casual partners	3/25	12.0			
Yes, with both	8/18	44.4			

^*^ Fisher's exact test

^**^ Adjusted by population and hand washing

^***^ aPR = adjusted prevalence ratios; CI = confidence intervals

## Discussion

Little is known about the impact of the socio-behavioral conditions of sexual minority and vulnerable groups on the acquisition of SARS-CoV-2 infection, such as adolescent MSM and TGW. To the best of our knowledge, no data on the prevalence of anti-SARS-CoV-2 antibodies in these populations have been published to date. The results from this study showed that approximately a fifth of the participants had anti-SARS-CoV-2 IgG or IgM antibodies during the first wave of COVID-19 in Brazil.

Comparing this result with prevalence rates found in the general population, or in other specific populations, it was observed a relatively high prevalence of anti-SARS-CoV-2 antibodies in AMSM and ATGW. A study performed in the general population in several Brazilian cities found prevalence rates of anti-SARC-CoV-2 antibodies of 1.6% (in May/2020) and 2.8% (in June/2020), varying considerably between regions of the country [22]. Another study conducted in Spain, from April to September 2020, with people infected with HIV, revealed a prevalence of anti-SARS-CoV-2 antibodies of 8.5% [23]. Studies carried out with children or adolescents have shown a variable prevalence of COVID-19. For example, a study that examined data from Departments of Health websites in six US states in 2020 found the prevalence in adolescents and young people to be around 1.3% - 2.2% [24]. A study that analyzed data from November 9 to December 9, 2020 (second wave), in Fortaleza, Brazil, found a prevalence of COVID-19 in adolescents of 29.2% distributed in IgM + IgG (9.7%), IgG (15.3%) and IgM (4.2%) [25]. Another study carried out with adolescents in England showed a seroprevalence of less than 10% until September-December 2020, where those aged 15 to 18 years increased to 23% [26].

The higher prevalence observed in comparison to other studies may be associated with several factors related to the different study designs or technical issues, such as the number of participants, the different antibody detection methodologies used, and the pandemic period at the study site. COVID-19, which is mainly transmitted person-to-person, also seems to have a high risk of spreading among these groups as evidenced by the high prevalence of anti-SARS-CoV-2 antibodies observed in this study.

The city of Salvador, in Bahia, is the most populous in the Northeast Region of Brazil and has high social inequality [27]. During the first wave of the pandemic, the city applied stricter lockdown restrictions than other cities in the country and had one of the higher rates of social isolation [28]. The AMSM and ATGW participants of this study showed high adherence to COVID-19 prevention measures and perception of SARS-CoV-2 infection risk. However, even in periods with a high number of hospitalizations in the city, when restrictions included the closing of services and limitations for social gatherings, most participants reported not wearing masks in public places, and maintaining sexual activity. This is in line with other studies showing the impact of MSM sexual activity during the pandemic [14]. Considering the widespread denialist discourses on the seriousness of COVID-19, especially from the Brazilian Federal Government [16], it is possible to hypothesize that gender- and sexuality-diverse youth could have dealt with conflicting messages about the pandemic. In addition, an increase in psychological distress due to home confinement, family conflicts, and restriction of social circles was observed during this time [15]. Therefore, the ability to manage self-care and protect from COVID-19 may have been hindered even in the presence of high knowledge and adherence to some protection measures [15].

Failure to adopt preventive behaviors such as wearing a mask in public places was associated with higher odds of having had contact with the virus during the first wave of the pandemic when the transmission rate was high. Studies have shown the importance of non-pharmacological measures in mitigating infection transmission [29]. The seroprevalence was associated with a belief in an easy cure for the disease. The perception of COVID-19 and its restriction measures is broad and encompasses individual, social, and scientific factors. In general, risk perception can vary substantially and be correlated with the adoption of preventive health behaviors as indicated by a study conducted in several countries worldwide [30].

Among the limitations of this study, due to its cross-sectional design, it was not possible to identify the temporal relationship between outcome and exposure variables, therefore its results must be interpreted cautiously. Nevertheless, the results are based on plausible associations, in line with current knowledge about the pandemic. In addition, sociodemographic data related to income and education, which could have improved the goodness-of-fit of the model, were not analyzed. We highlight the convenience sampling of this study as participants are linked to a PrEP cohort study, and are generally more exposed to sexual intercourse.

## Conclusion

The high prevalence of SARS-CoV-2 among gender and sexual minority youth seems to be informed by behaviors and attitudes that contrast with public health measures and the potential severity of the disease when vaccination was still not available. The vulnerability of LGBT communities to the pandemic and the results should be considered by public health decision-makers in designing health education interventions that are culturally acceptable and people-centered, i.e., addressing individual preferences, needs, and values.

## Data Availability

The datasets used and/or analyzed during the current study are available from the corresponding author on reasonable request.
